# Comparative evaluation of ChatGPT, DeepSeek, and Doubao for pediatric IgA vasculitis patient education: content quality, reliability, readability, understandability, and actionability

**DOI:** 10.3389/fmed.2026.1974214

**Published:** 2026-10-21

**Authors:** Ximing Fei, Jiao Zhang, Qiuye Li, Jianhui Yang

**Affiliations:** 1 The Second Clinical Medical College, Guizhou University of Traditional Chinese Medicine, Guiyang, Guizhou, China; 2 Department of Pediatrics, The Second Affiliated Hospital of Guizhou University of Traditional Chinese Medicine, Guiyang, Guizhou, China

**Keywords:** immunoglobulin A vasculitis, large language models, patient education, readability, understandability and actionability

## Abstract

**Background:**

Large language model (LLM) chatbot platforms are increasingly used for health information, but their performance for pediatric immunoglobulin A vasculitis (IgAV) patient education is unclear.

**Objective:**

To compare ChatGPT, DeepSeek, and Doubao across content completeness, quality and reliability, readability, understandability, and actionability.

**Methods:**

Thirty-two standardized English questions were submitted to ChatGPT, DeepSeek, and Doubao on August 9, 2026, using a new conversation for each question and retaining the first complete response (96 responses). Two reviewers independently performed the original assessment, with third-reviewer adjudication. GQS, mDISCERN, QAMAI, six readability metrics, PEMAT-P, and ICC(2,1) were used. During revision, a question-specific clinical checklist was established and applied to all archived answers. The research team completed the revision scoring and confirmed the final omission results.

**Results:**

Clinically relevant information omissions occurred in 16/96 responses (16.67%): 0/32 for ChatGPT, 5/32 for DeepSeek, and 11/32 for Doubao. Retained ratings identified outdated medical advice in 2/96 responses (2.08%) and no real-time information errors or hallucinations. At least one recorded content deficiency occurred in 18/96 responses (18.75%). Original ICC(2,1) values ranged from 0.767 to 0.954. Overall differences were significant for GQS, mDISCERN, QAMAI, PEMAT-U, and PEMAT-A (all *P* < .01), but not for readability metrics. Bonferroni-adjusted contrasts showed lower ARI, FKGL, CLI, and SMOG scores for DeepSeek than ChatGPT. DeepSeek had the highest PEMAT-U score; ChatGPT had the highest PEMAT-A score.

**Conclusions:**

Performance varied by evaluation domain, and clinically relevant omissions remained a safety concern. The omission findings reflect criteria established during revision and do not establish a validated platform safety ranking. LLM platforms may supplement patient education but should not replace professional medical review.

## 1 Introduction

Immunoglobulin A vasculitis (IgAV), formerly known as Henoch-Schönlein purpura, is one of the most common systemic small-vessel vasculitides in children and typically involves the skin, joints, gastrointestinal tract, and kidneys. A recent review summarized advances in IgAV treatment and management (1), while recent Japanese, UK, IPNA, and KDIGO guidelines and recommendations have further standardized diagnosis, risk-stratified treatment, and follow-up (2–5). IgAV nephritis (IgAVN) is a major determinant of long-term outcomes; persistent proteinuria, impaired kidney function, and high-risk histopathologic findings warrant structured renal monitoring and long-term follow-up (6–9). Severe gastrointestinal manifestations are also important clinical risks in pediatric IgAV (10). Accordingly, recognition of warning signs, monitoring for renal involvement, treatment safety, and follow-up planning are key components of patient education.

Digital health information has become an important complement to conventional clinician-patient communication. With the rapid development of generative artificial intelligence and LLMs, the public can obtain interactive health information through conversational interfaces. Recent scoping reviews of patient education have shown that LLMs are increasingly used for patient-facing question answering and educational material generation (11, 12), while systematic reviews and broader medical studies continue to highlight concerns regarding accuracy, transparency, consistency, and clinical safety (13–15). In real-world patient-question settings, AI-generated answers may also differ substantially from physician responses in both content and communication style (16). Cross-specialty studies have further shown heterogeneous platform performance in pediatric anesthesia, peritoneal dialysis, and hypertension (17–19), while studies in oral lichen planus and prostate cancer have reported limitations in readability, completeness, or source transparency (20, 21).

The clinical value of medical LLM responses cannot be judged solely by surface fluency or factual plausibility. Because LLMs generate text probabilistically, they may produce unsupported statements or factual errors, or omit warning signs, monitoring requirements, and indications for medical attention that directly affect patient decision-making. Recent systematic reviews, real-world patient-question comparisons, and pediatric anesthesia studies have shown that LLM responses may be well structured and of reasonable overall quality yet still contain errors, omissions, or inadequate sourcing (13, 16, 17). Evaluation of patient-facing LLMs therefore requires simultaneous consideration of accuracy, completeness, reliability, and safety-related deficiencies.

Patient-education information should also be readable, understandable, and actionable. QAMAI provides a structured framework for assessing AI-generated medical information across accuracy, clarity, relevance, completeness, source provision, and usefulness (22). Dental studies have shown that LLMs may perform differently across quality, understandability, and actionability (23). With respect to readability, recent cross-disciplinary studies suggest that rewriting or prompting for a lower reading level can reduce linguistic complexity in some patient-education materials (24–27), although dermatology and orthopedic studies indicate that such improvements are not always consistent or sufficient (28, 29). Studies in pharmacy, urology, and colorectal cancer likewise emphasize the need to assess whether information is understandable, supports concrete actions, and preserves medical quality (30–32). Readability, understandability, and actionability are therefore related but noninterchangeable constructs.

Multidimensional cross-platform evaluations of LLMs specifically for IgAV patient education remain limited. This study compared standardized IgAV responses generated by ChatGPT, DeepSeek, and Doubao across content accuracy and completeness, quality and reliability, readability, understandability, and actionability, and quantified interrater reliability to evaluate their potential value and limitations as adjunctive sources of pediatric IgAV patient education.

## 2 Methods

### 2.1 Study design

This cross-sectional, cross-platform comparative study evaluated pediatric IgAV patient-education responses generated by ChatGPT, DeepSeek, and Doubao in their default user environments. The study focused on the end-to-end patient-education performance delivered to ordinary users rather than on direct comparisons of underlying language-model capabilities under standardized technical parameters. The workflow comprised patient-question development and screening, standardized prompting and response collection, human assessment, automated readability assessment, and statistical analysis (Figure 1). No patients or other human participants were recruited, and no identifiable personal health information was used. The study protocol was reviewed and approved by the Ethics Committee of the Second Affiliated Hospital of Guizhou University of Traditional Chinese Medicine (approval no. LW20260808; August 8, 2026).

**Figure 1:**
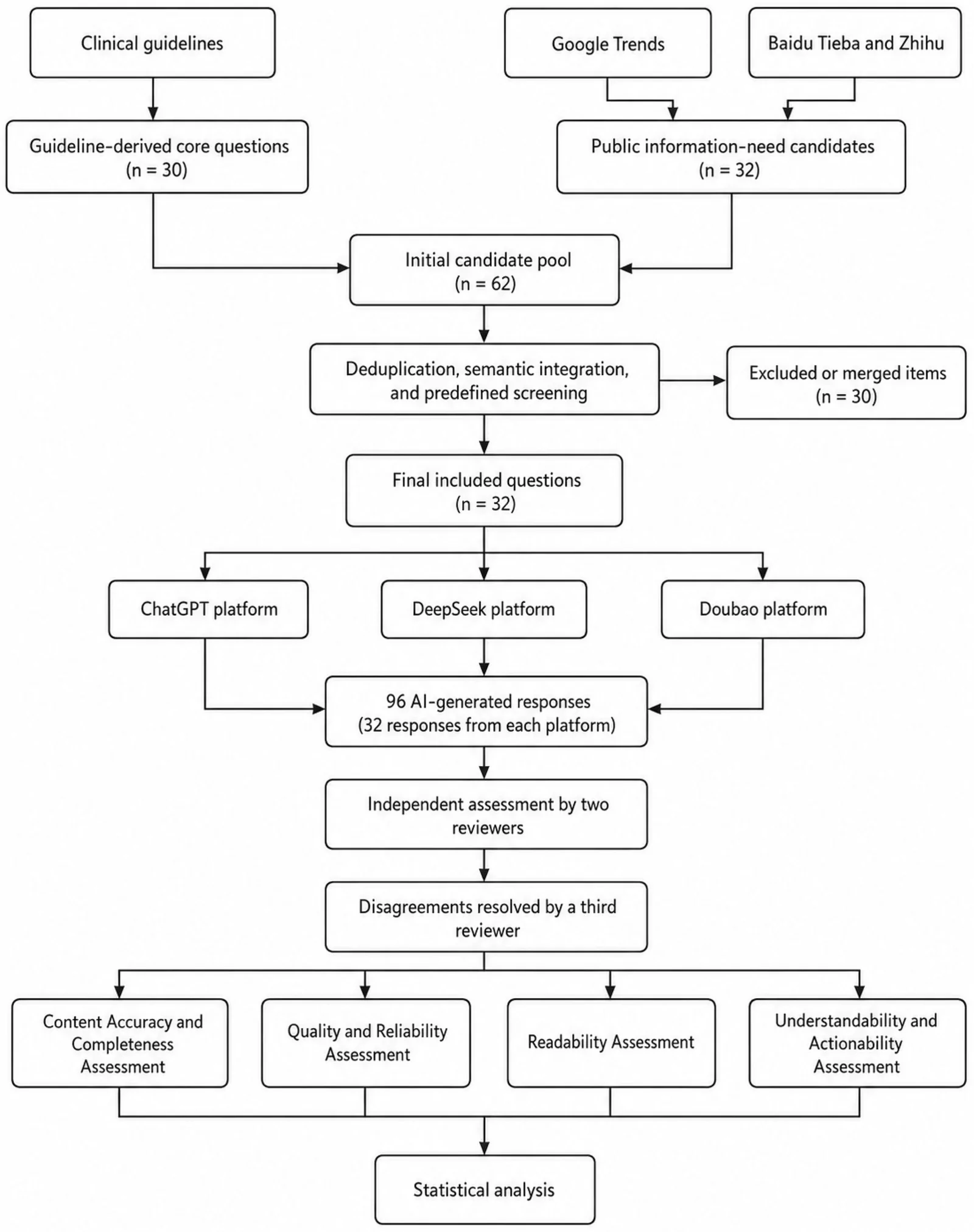
Workflow for question screening, LLM response generation, independent assessment by two primary reviewers, adjudication by a third reviewer, multidimensional evaluation, and statistical analysis.

### 2.2 Development and screening of patient-education questions

A guideline-led approach, supplemented by cross-checking of public information needs, was used to develop the pediatric IgAV patient-education question set. Recent pediatric IgAV and IgAVN guidelines and clinical practice recommendations (2–5), together with studies on renal outcomes, treatment, and gastrointestinal risk (6, 8–10), were used to identify content directly relevant to patient and caregiver decision-making. These concepts were converted into standardized, single-topic questions covering disease concepts, causes and organ involvement, clinical manifestations and warning signs, diagnosis and differential diagnosis, renal screening and severity assessment, treatment and medication monitoring, prognosis and follow-up, and home management, yielding 30 guideline-derived questions.

To supplement and cross-check public information needs, Google Trends, Zhihu, and Baidu Tieba were searched on August 9, 2026. Google Trends was set to Worldwide for the period from August 9, 2021, to August 9, 2026, using “IgA vasculitis” as the search term and “All categories” as the category; one related query was identified through the Related queries module. Zhihu and Baidu Tieba were searched using the Chinese keyword “IgA血管炎,” yielding 20 and 11 candidate items, respectively. Questions from Chinese-language sources were standardized into English by two medically trained researchers proficient in English, and a third researcher independently reviewed and confirmed semantic consistency. Only publicly accessible questions or information needs from social media were recorded; usernames, account information, and other personally identifiable information were not collected.

The guideline, Google Trends, and social-media sources yielded 62 candidate items. Prespecified procedures were used for deduplication, semantic integration, and screening: exact duplicates were removed; highly similar items were merged; nonguideline items that substantially overlapped with a guideline-derived question were represented by the corresponding guideline question; and items unrelated to IgAV, overly broad or vague, overly specialized, potentially misleading, or requiring highly individualized clinical judgment were excluded. The final set comprised 32 standardized patient-education questions, including 30 that retained the core content of guideline-derived questions and 2 nonguideline questions, both addressing differential diagnosis. The final 32 English questions are provided in Supplementary Table S1, and the sources and screening process of candidate questions are illustrated in Figure 1.

### 2.3 Chatbot platform selection, operating environment, and response collection

ChatGPT, DeepSeek, and Doubao were selected because all three are conversational generative-AI platforms directly accessible to the general public and can be used for health-information queries. The unit of evaluation was the real-world conversational service delivered to ordinary users at the time of data collection rather than a separately deployed underlying foundation model.

All responses were collected on August 9, 2026. The same 32 standardized English questions were submitted verbatim to ChatGPT, DeepSeek, and Doubao, yielding 96 responses, with 32 responses per platform. Each platform was used in the default web-based conversational environment available to users on the collection date. To minimize contextual carryover from preceding questions, each question was submitted in a separate new conversation. Only the first complete response was recorded; no follow-up prompting, regeneration, or manual editing was performed.

No additional instructions such as “search the web,” “do not search the web,” or “provide references” were sent to any platform, and the researchers did not manually enable or disable default information-retrieval functions. The responses therefore reflect the end-to-end output delivered by each platform in its default user environment at the time of data collection. Because platforms may differ in underlying model routing, retrieval augmentation, system prompts, and product-level functions, the findings were not used to infer or rank the independent capabilities of their underlying foundation models.

Platform version information was reported only when it could be directly confirmed from the user interface or platform records on the date of data collection. No inference was made regarding underlying model names or internal routing versions that could not be reliably verified by the user. All questions, response dates, and platform information were documented to improve traceability. At data collection, the ChatGPT interface displayed GPT-5.6. For DeepSeek and Doubao, the research team did not obtain user-facing information that could independently verify the specific underlying model versions used on that date, and therefore no further base-model designation was assigned. This standardized prompting and reporting strategy is consistent with recent calls for reproducibility in medical LLM evaluations (12, 13).

### 2.4 Reviewer assessment and adjudication of disagreements

For the original assessment, two graduate students with pediatric clinical training independently evaluated all LLM responses. Before formal scoring, both reviewers completed standardized training and calibration on the scoring rules for each assessment instrument and the criteria for identifying content deficiencies. Disagreements were adjudicated by a senior physician with 5 years of pediatric clinical experience. Interrater reliability was assessed using a two-way random-effects model with absolute agreement and a single-measure intraclass correlation coefficient [ICC(2,1)]. Reviewers were not blinded to platform identity. The revision-stage omission scoring is described in Section 2.5. The original ICC estimates were retained and were not recalculated for the revised omission codes.

### 2.5 Assessment of content accuracy and completeness

Content accuracy and completeness were assessed primarily against recent pediatric IgAV and IgAVN clinical guidelines and international recommendations (2–5). For questions involving renal outcomes, treatment, or gastrointestinal risk, recent disease-management studies were additionally consulted (8, 10). Two reviewers examined disease facts, warning signs, investigations and monitoring, treatment recommendations, medication safety, and follow-up information, and identified four prespecified categories of content deficiency: (1) important information omission, defined as the absence of key information explicitly emphasized in current guidelines or recommendations and necessary for a safe and complete answer to the question; (2) outdated medical advice, defined as advice inconsistent with current major guidelines or clinical practice; (3) errors related to real-time information updates; and (4) hallucinations, defined as fabricated, unsupported, or clearly falsifiable information presented as fact. When current guidelines differed within a reasonable range, use of any evidence-supported recommendation was not classified as an error. Disputed judgments were adjudicated by the third reviewer. This framework was informed by recent studies of patient health advice and patient-education LLMs that evaluated factual errors, information omissions, and hallucination risks (13, 16, 17). Each deficiency category was summarized by the number and proportion of responses in which it occurred.

During revision on October 3, 2026, a written question-specific clinical reference checklist was formalized for the 32 questions (Supplementary Table S2). The original assessment had no written question-specific checklist. The appendix therefore documents a revision-stage operational definition, not a standard prespecified before the original evaluation. It contains 97 elements derived from Chinese clinical guidelines, SCI/SCIE-journal guidelines, formal guideline digests, or consensus practice recommendations; the element selection and safety thresholds are study operational rules rather than a validated scale.

The archived answers were evaluated separately against the corresponding question criteria. Coverage was coded as adequate, partial, missing, incorrect, or conditionally not applicable. Semantically equivalent plain-language advice was accepted. The response-level code was 1 only when an applicable absent component was essential to safe caregiver decisions, including care-seeking, treatment safety, recognition of organ involvement, or required monitoring and follow-up. Otherwise, the code was 0. Partial coverage did not automatically qualify; incorrect advice was recorded separately from missing information. Multiple qualifying gaps in one response counted as one omission event. Content from other questions was not used to complete an answer.

Codex (OpenAI, GPT-6, accessed through ChatGPT Work on October 3, 2026) generated the initial checklist-based codes and 291 element-level records for the 96 archived responses. Each response-level code was linked to element IDs, an explanation, a verbatim source excerpt, and guideline locators. The research team subsequently completed the revision scoring and confirmed 16 responses with clinically relevant omissions on October 4, 2026. These final omission scores replace the earlier omission results in Table 1. Earlier scores are retained in the Supplementary Dataset for traceability. The ratings for outdated medical advice, real-time information errors, and hallucinations were retained from the original assessment and were not comprehensively rescored in this omission revision. Any content deficiency was recomputed for each response as the union of its final omission score and these three retained category ratings. The revision did not alter the quality scores or original ICC estimates, and no new independent two-reviewer procedure or third-reviewer adjudication is asserted for the final omission codes.

**Table 1:** Content deficiencies in responses generated by ChatGPT, DeepSeek, and Doubao after revision-stage omission scoring.

Content deficiency	ChatGPT (*n* = 32)	DeepSeek (*n* = 32)	Doubao (*n* = 32)	Total (*n* = 96)
Clinically relevant information omissions	0 (0.00)	5 (15.63)	11 (34.38)	16 (16.67)
Outdated medical advice	0 (0.00)	1 (3.13)	1 (3.13)	2 (2.08)
Real-time information errors	0 (0.00)	0 (0.00)	0 (0.00)	0 (0.00)
Hallucinations	0 (0.00)	0 (0.00)	0 (0.00)	0 (0.00)
Any content deficiency	0 (0.00)	6 (18.75)	12 (37.50)	18 (18.75)

Data are presented as *n* (%). The denominator is 32 per platform and 96 overall. Omission counts reflect the final revision scoring using the checklist established during revision. The other three category ratings were retained from the original assessment. Any content deficiency denotes their response-level union with the final omission score; a response is counted once. Earlier omission codes are retained in the Supplementary Dataset. LLM, large language model.

### 2.6 Quality and reliability assessment

The Global Quality Score (GQS), modified DISCERN (mDISCERN), and Quality Analysis of Medical Artificial Intelligence (QAMAI) were used to assess response quality and reliability. GQS is an overall 1- to 5-point rating, with higher scores indicating better overall quality and usefulness. mDISCERN comprises five binary items addressing clarity, source reliability, balanced and objective presentation, provision of additional sources, and acknowledgment of uncertainty, with a total score of 0 to 5; higher scores indicate greater reliability. GQS and mDISCERN have been used in recent specialty-specific evaluations of LLM-generated patient health information (17–20). QAMAI assesses six domains—accuracy, clarity, relevance, completeness, source/reference provision, and usefulness—each scored from 1 to 5, for a total score of 6 to 30; higher scores indicate better overall quality of AI-generated medical information (22).

### 2.7 Readability assessment

Readability of all English responses was evaluated using the ReadabilityFormulas.com online scoring system (accessed August 10, 2026). The Automated Readability Index (ARI), Flesch Reading Ease Score (FRES), Gunning Fog Index (GFI), Flesch-Kincaid Grade Level (FKGL), Coleman-Liau Index (CLI), and Simple Measure of Gobbledygook (SMOG) were calculated. These metrics have been used in recent LLM patient-education studies to assess reading grade level and text complexity (17, 21, 24, 26), with similar approaches reported in dermatology and orthopedic patient-education research (28, 29). With the exception of FRES, higher scores generally indicate a higher required reading grade level or greater linguistic complexity; higher FRES values indicate easier reading. All metrics were calculated directly from the first original English response generated by each platform, without translation or manual linguistic editing.

### 2.8 Understandability and actionability assessment

The Patient Education Materials Assessment Tool for Printable Materials (PEMAT-P) was used to evaluate understandability (PEMAT-U) and actionability (PEMAT-A). Applicable items were scored according to the tool instructions and converted to percentages; higher scores indicate that materials are easier to understand or more clearly support identification of concrete actions, respectively. Recent studies in hypertension, dentistry, pharmacy, and urology have used PEMAT-P to evaluate LLM outputs and have shown that understandability and actionability may diverge (19, 23, 30, 31).

### 2.9 Statistical analysis

GraphPad Prism 9.5.1 was used for normality testing, between-platform comparisons, and figure generation, and IBM SPSS Statistics 27.0 was used for interrater reliability analysis. Continuous-variable distributions were assessed using the Shapiro–Wilk test. Because the same 32 questions were submitted to each of the three platforms, between-platform comparisons were treated as paired repeated-measures data. GQS, mDISCERN, QAMAI, PEMAT-U, and PEMAT-A are presented as median and interquartile range [median (Q1, Q3)] and were compared overall using the Friedman test; pairwise comparisons used Dunn's multiple-comparisons test on within-question ranks. Readability metrics are presented as mean (SD) and were compared using repeated-measures one-way analysis of variance; sphericity was not assumed, and the Geisser-Greenhouse correction was applied. The three prespecified platform contrasts for each readability metric used paired comparisons with Bonferroni adjustment, regardless of the omnibus test result. Multiplicity adjustment was applied to the three platform contrasts within each metric; no correction across the 11 distinct outcomes was applied. Interrater reliability between the two primary reviewers was estimated using a two-way random-effects model, absolute-agreement definition, single-measure intraclass correlation coefficient [ICC(2,1)] with 95% confidence intervals. All tests were two-sided, and *P* < .05 was considered statistically significant.

Final omission scores and all recorded content deficiencies were summarized as counts and proportions, using denominators of 32 per platform and 96 overall. A response with multiple qualifying omissions counted once. Any content deficiency counted a response once when any of the four recorded category codes was 1. Changes from the earlier omission codes were retained at response level in the Supplementary Dataset. No new omission-code ICC or inferential platform comparison was calculated for this revision-stage analysis.

## 3 Results

### 3.1 Question screening and response generation

The initial candidate pool contained 62 items: 30 guideline/recommendation-derived questions, 1 Google Trends related query, 20 Zhihu items, and 11 Baidu Tieba items. After item-level screening and integration, 32 standardized questions were retained. Thirty were derived from guidelines/recommendations, and 2 were retained from Zhihu; both addressed differential-diagnosis information needs not sufficiently covered by the guideline-derived questions. No Google Trends or Baidu Tieba item remained as an independent final question. Of the remaining 30 candidate items, 8 were merged because of semantic overlap with guideline-derived questions; no exact duplicates were identified. A further 22 items were excluded: 6 were unrelated to IgAV, 8 were overly broad or vague, 2 were overly specialized, 5 were potentially misleading, and 1 required highly individualized clinical judgment. The final 32 questions were submitted to ChatGPT, DeepSeek, and Doubao, yielding 96 responses, 32 per platform (Figure 1).

### 3.2 Content accuracy and completeness

In the revised dataset, clinically relevant information omissions occurred in 16/96 responses (16.67%): 0/32 (0.00%) from ChatGPT, 5/32 (15.63%) from DeepSeek, and 11/32 (34.38%) from Doubao. Retained original ratings identified outdated medical advice in 2/96 responses (2.08%), including 1/32 (3.13%) from DeepSeek and 1/32 (3.13%) from Doubao. No real-time information errors or hallucinations were recorded. The two responses with outdated medical advice did not overlap with the 16 omission-positive responses. Therefore, at least one recorded content deficiency occurred in 18/96 responses (18.75%): 0/32 (0.00%) from ChatGPT, 6/32 (18.75%) from DeepSeek, and 12/32 (37.50%) from Doubao (Table 1). The final omission scores replace the earlier scores; 27 response codes changed, comprising 12 earlier positive scores changed to 0 and 15 earlier negative scores changed to 1. Supporting records are included in the Supplementary Dataset.

### 3.3 Interrater reliability

Across expert-rated measures, single-measure ICC(2,1) values for the two primary reviewers ranged from 0.767 to 0.954. ICC(2,1) was 0.822 (95% CI 0.744–0.878) for GQS, 0.839 (0.768–0.890) for mDISCERN, 0.954 (0.931–0.969) for QAMAI, 0.767 (0.671–0.839) for PEMAT-U, and 0.802 (0.718–0.863) for PEMAT-A; all *P* < .01 (Table 2).

**Table 2:** Interrater reliability between the two primary reviewers: single-measure intraclass correlation coefficients [ICC(2,1)].

Assessment instrument	ICC	95% CI	*P* value
GQS	0.822	0.744–0.878	<.01
mDISCERN	0.839	0.768–0.890	<.01
QAMAI	0.954	0.931–0.969	<.01
PEMAT-U	0.767	0.671–0.839	<.01
PEMAT-A	0.802	0.718–0.863	<.01

ICC(2,1) denotes a single-measure intraclass correlation coefficient based on a two-way random-effects model and an absolute-agreement definition; CI, confidence interval; GQS, Global Quality Score; QAMAI, Quality Analysis of Medical Artificial Intelligence; PEMAT-U, understandability; PEMAT-A, actionability.

### 3.4 Quality and reliability

Overall differences among platforms were significant for GQS, mDISCERN, and QAMAI (all *P* < .01; Tables 3, 6; Figure 2). Median GQS scores were 4.00 (4.00, 4.00) for ChatGPT, 4.00 (3.00, 4.00) for DeepSeek, and 3.00 (3.00, 4.00) for Doubao; ChatGPT scored higher than Doubao (Dunn-adjusted *P* = .0444). Median mDISCERN scores were 4.00 (4.00, 5.00), 4.00 (4.00, 5.00), and 4.00 (3.00, 4.00), respectively; ChatGPT scored higher than Doubao (*P* = .0179). Median QAMAI scores were 21.00 (19.75, 23.00), 23.00 (22.00, 23.00), and 20.00 (19.00, 21.00), respectively; both DeepSeek (*P* < .0001) and ChatGPT (*P* = .0216) scored higher than Doubao, whereas DeepSeek and ChatGPT did not differ significantly (*P* = .1581).

**Table 3:** Comparison of response quality and reliability across ChatGPT, DeepSeek, and Doubao.

Measure	ChatGPT	DeepSeek	Doubao	*P* value
GQS, median (IQR)	4.00 (4.00,4.00)	4.00 (3.00,4.00)	3.00 (3.00,4.00)	<.01
mDISCERN, median (IQR)	4.00 (4.00,5.00)	4.00 (4.00,5.00)	4.00 (3.00,4.00)	<.01
QAMAI, median (IQR)	21.00 (19.75,23.00)	23.00 (22.00,23.00)	20.00 (19.00,21.00)	<.01

Data are presented as median (IQR). Overall comparisons among the three platforms were performed using the Friedman test. Dunn-adjusted pairwise *P* values are reported in Table 6.

**Figure 2:**
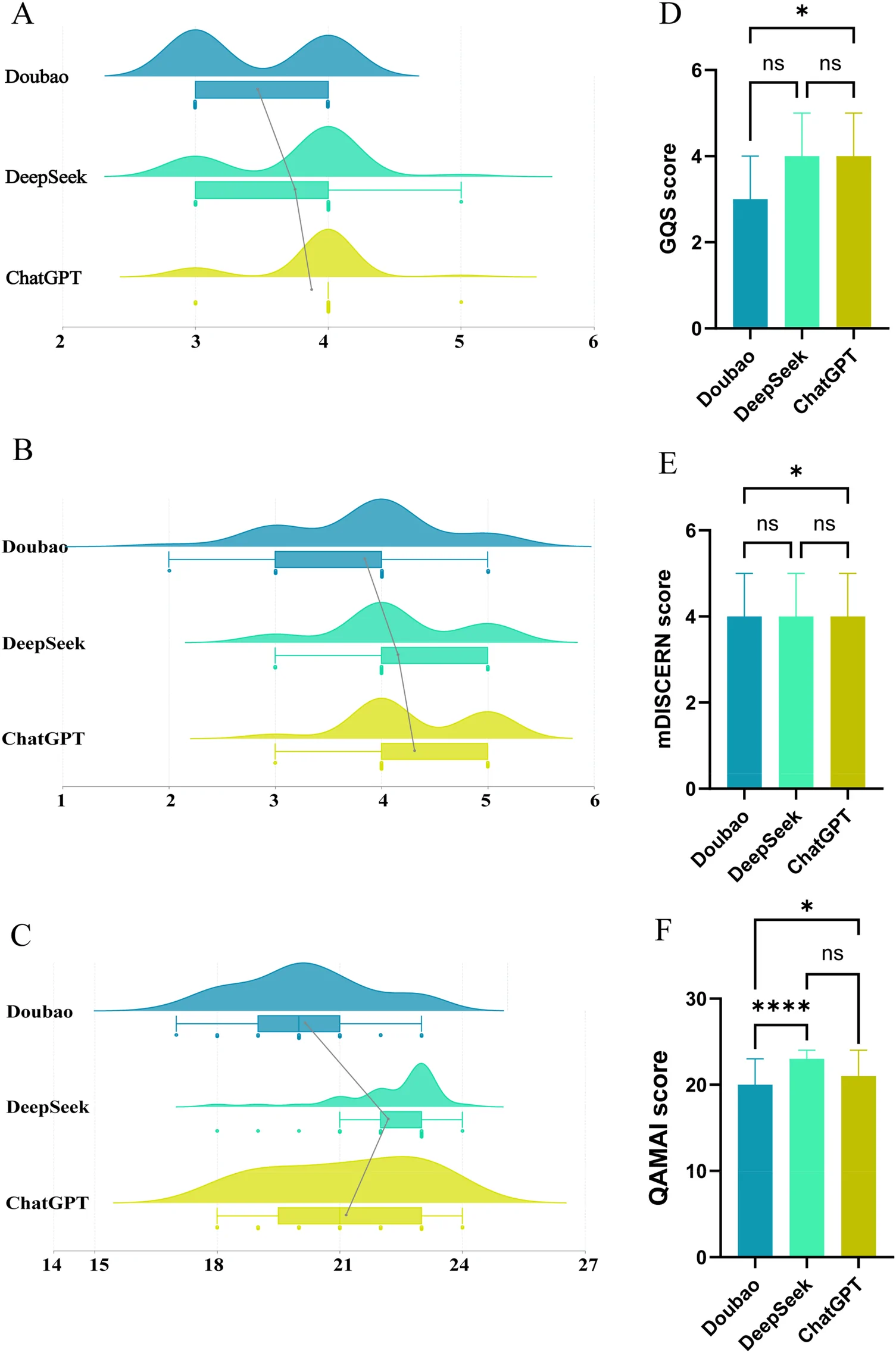
Comparison of response quality and reliability across ChatGPT, DeepSeek, and Doubao. Panel **(A–C)** show the distributions of GQS, mDISCERN, and QAMAI scores, respectively; panel **(D–F)** show the corresponding between-platform comparisons. Overall comparisons for GQS, mDISCERN, and QAMAI were performed using the Friedman test, and significance markers indicate multiplicity-adjusted pairwise comparisons. ns, not significant; *P < .05; ****P < .0001.

### 3.5 Readability

Across all 96 responses, mean ARI was 15.65 ± 3.23, FRES 25.99 ± 15.71, GFI 14.84 ± 2.66, FKGL 14.21 ± 2.94, CLI 16.22 ± 2.77, and SMOG 12.39 ± 2.17. No significant overall platform effect was observed for ARI (Geisser-Greenhouse-corrected *P* = .0547), FRES (*P* = .1150), GFI (*P* = .6123), FKGL (*P* = .0675), CLI (*P* = .0848), or SMOG (*P* = .0988) (Table 4; Figures 3, 4). The prespecified ChatGPT-DeepSeek contrasts, assessed independently of the omnibus decision and Bonferroni adjusted within each metric, showed lower scores for DeepSeek on ARI (adjusted *P* = .0193), FKGL (adjusted *P* = .0227), CLI (adjusted *P* = .0235), and SMOG (adjusted *P* = .0427). The remaining readability contrasts were not significant after adjustment (Table 6).

**Table 4:** Comparison of readability metrics across ChatGPT, DeepSeek, and Doubao.

Readability metric	ChatGPT, mean (SD)	DeepSeek, mean (SD)	Doubao, mean (SD)	*P* value
ARI	16.63 (2.55)	15.00 (2.91)	15.33 (3.93)	0.0547
FRES	23.53 (13.25)	29.16 (15.46)	25.28 (18.02)	0.1150
GFI	14.88 (2.57)	14.58 (2.27)	15.05 (3.13)	0.6123
FKGL	14.98 (2.28)	13.61 (2.73)	14.05 (3.57)	0.0675
CLI	16.71 (2.36)	15.64 (2.66)	16.31 (3.20)	0.0848
SMOG	12.98 (1.79)	11.95 (1.86)	12.24 (2.69)	0.0988

Data are presented as mean (SD). Overall comparisons among the three platforms were performed using repeated-measures one-way analysis of variance, with Geisser-Greenhouse correction for all six metrics. Bonferroni-adjusted pairwise *P* values are reported in Table 6.

**Figure 3:**
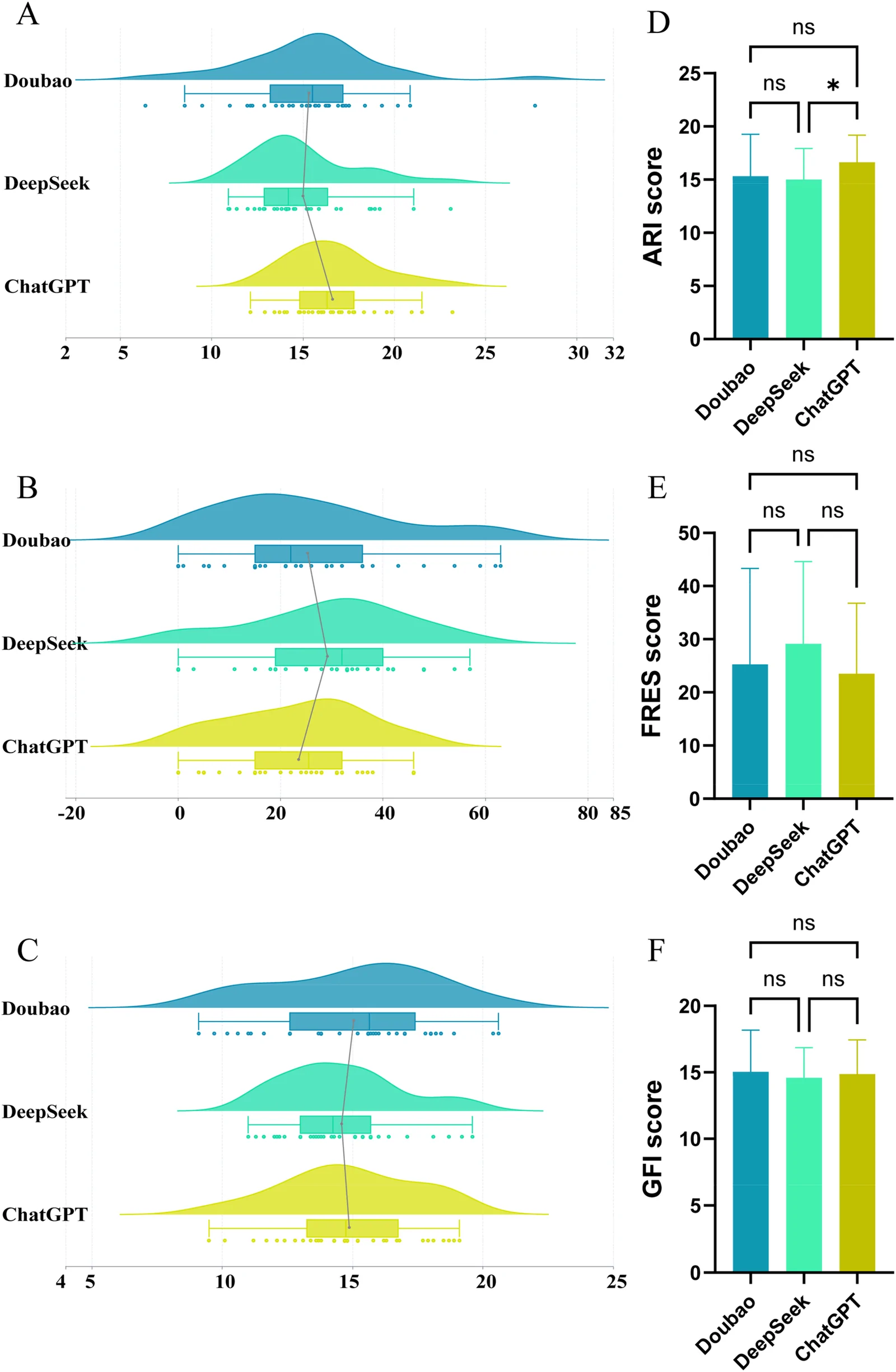
Comparison of ARI, FRES, and GFI readability metrics across the three platforms. Panel **(A–C)** show the distributions of ARI, FRES, and GFI scores, respectively; panel **(D–F)** show the corresponding between-platform comparisons. Overall comparisons were performed using repeated-measures one-way analysis of variance, and prespecified pairwise comparisons were Bonferroni adjusted. ns, not significant; *P < .05.

**Figure 4:**
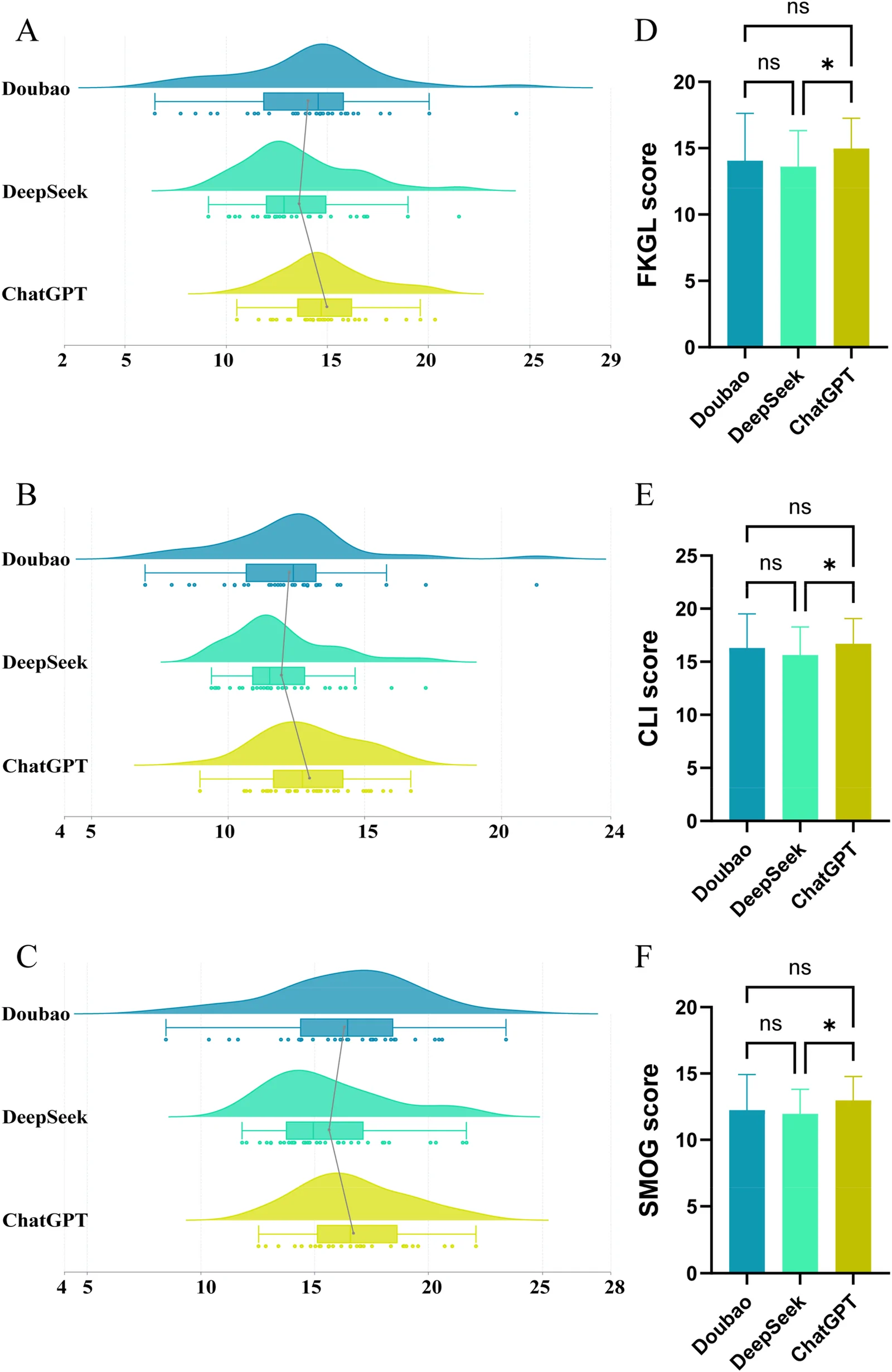
Comparison of FKGL, CLI, and SMOG readability metrics across the three platforms. Panel **(A–C)** show the distributions of FKGL, CLI, and SMOG scores, respectively; panel **(D–F)** show the corresponding between-platform comparisons. Overall comparisons were performed using repeated-measures one-way analysis of variance, and prespecified pairwise comparisons were Bonferroni adjusted. ns, not significant; *P < .05.

### 3.6 Understandability and actionability

Overall differences among platforms were significant for both PEMAT-U and PEMAT-A (both *P* < .01; Tables 5, 6; Figure 5). Median PEMAT-U scores were 67.00 (57.75, 70.00) for ChatGPT, 76.50 (69.00, 77.00) for DeepSeek, and 56.50 (50.00, 67.00) for Doubao. DeepSeek scored higher than ChatGPT (Dunn-adjusted *P* = .0179) and Doubao (*P* < .0001), while ChatGPT and Doubao did not differ significantly (*P* = .4517). Median PEMAT-A scores were 60.00 (40.00, 80.00), 50.00 (35.00, 60.00), and 40.00 (20.00, 60.00), respectively. ChatGPT scored higher than DeepSeek (*P* = .0179) and Doubao (*P* = .0373), while DeepSeek and Doubao did not differ significantly (*P* = 1.0000). These patterns distinguish understandability from actionability.

**Table 5:** Comparison of response understandability and actionability across ChatGPT, DeepSeek, and Doubao.

Measure	ChatGPT	DeepSeek	Doubao	*P* value
PEMAT-U, median (IQR)	67.00 (57.75,70.00)	76.50 (69.00,77.00)	56.50 (50.00,67.00)	<.01
PEMAT-A, median (IQR)	60.00 (40.00,80.00)	50.00 (35.00,60.00)	40.00 (20.00,60.00)	<.01

Data are presented as median (IQR). Overall comparisons among the three platforms were performed using the Friedman test. Dunn-adjusted pairwise *P* values are reported in Table 6. PEMAT-U, understandability; PEMAT-A, actionability.

**Table 6:** Adjusted *P* values for pairwise comparisons between platforms.

Measure	ChatGPT vs. DeepSeek	ChatGPT vs. Doubao	DeepSeek vs. Doubao
GQS	1.0000	0.0444	0.2745
mDISCERN	0.9519	0.0179	0.2404
QAMAI	0.1581	0.0216	<.0001
PEMAT-U	0.0179	0.4517	<.0001
PEMAT-A	0.0179	0.0373	1.0000
ARI	0.0193	0.3452	1.0000
FRES	0.0583	1.0000	0.5848
GFI	1.0000	1.0000	1.0000
FKGL	0.0227	0.5092	1.0000
CLI	0.0235	1.0000	0.5731
SMOG	0.0427	0.5716	1.0000

*P* values are adjusted across the three platform contrasts within each measure. GQS, mDISCERN, QAMAI, PEMAT-U, and PEMAT-A use the Friedman test with Dunn's rank-based multiple comparisons. Readability measures use prespecified paired comparisons after repeated-measures ANOVA with Bonferroni adjustment; the omnibus *P* value is Geisser-Greenhouse corrected where required. Pairwise testing was not contingent on a significant omnibus result. Values below.0001 are shown as <.0001.

**Figure 5:**
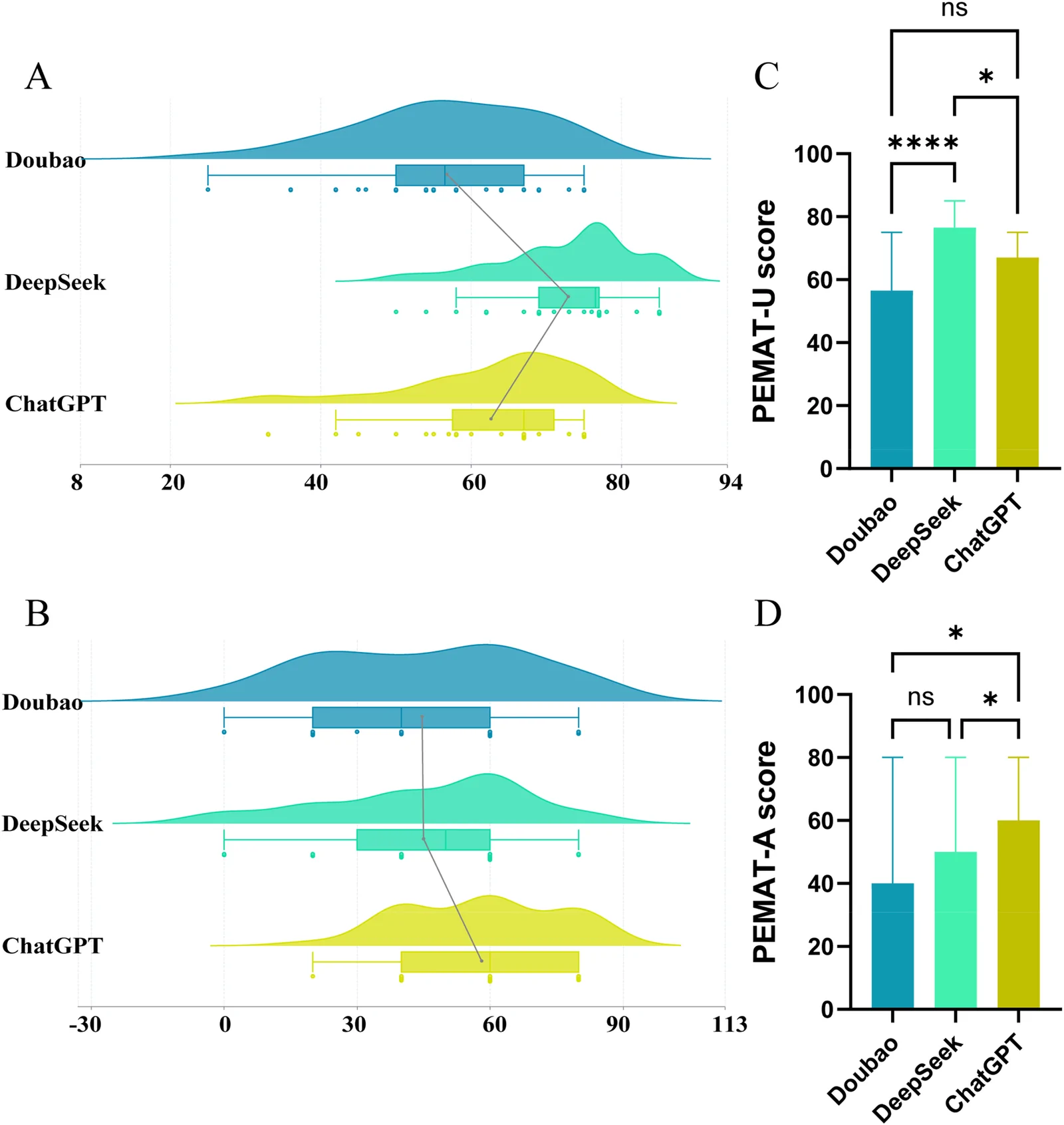
Comparison of PEMAT-P understandability and actionability scores across ChatGPT, DeepSeek, and Doubao. Panel **(A and B)** show the distributions of PEMAT-U and PEMAT-A scores, respectively; Panel **(C and D)** show the corresponding between-platform comparisons. Overall comparisons were performed using the Friedman test, and significance markers indicate multiplicity-adjusted pairwise comparisons. ns, not significant; *P < .05; ****P < .0001.

## 4 Discussion

### 4.1 Principal findings

This study conducted a multidimensional comparison of pediatric IgAV patient-education information generated by ChatGPT, DeepSeek, and Doubao in their default user environments. The principal findings were that clinically relevant information omissions were more common than hallucinations; relative performance across GQS, mDISCERN, and QAMAI was not uniform; all six readability metrics indicated substantial reading burden without a significant overall platform effect; DeepSeek had the highest understandability score, whereas ChatGPT had the highest actionability score. These findings indicate that the performance of patient-facing LLM platforms is strongly domain dependent. Accordingly, the results primarily reflect the end-to-end patient-education experience delivered to ordinary users by the three platforms at the August 2026 data-collection time point and should not be interpreted as a direct ranking of their underlying foundation models.

### 4.2 Information omission as a clinically important safety dimension

The revised assessment identified clinically relevant omissions in 16.67% of responses (0 ChatGPT, 5 DeepSeek, and 11 Doubao responses). The retained original ratings identified no hallucinations meeting the study definition. For patient education, safety depends on completeness of warning signs, renal monitoring, medication restrictions, and indications for timely medical attention as well as factual correctness. These elements are emphasized in pediatric IgAV and IgAVN guidance (3–5, 8); severe abdominal pain, gastrointestinal bleeding, and persistent vomiting likewise warrant timely medical assessment (10). The absence of qualifying omissions in the 32 ChatGPT responses does not establish that they were fully correct or clinically safe.

For example, the Doubao response to Question 19, which asked whether early glucocorticoid treatment can prevent IgAV nephritis, was coded as having a clinically relevant omission in the response-level dataset. It correctly stated that prophylactic glucocorticoids should not be used solely to prevent nephritis, but gave no advice about subsequent urine monitoring. KDIGO recommends urinary monitoring at presentation and at least monthly for six months, even though glucocorticoids are not supported for primary prevention of nephritis (5). Without that distinction, a caregiver may not arrange the recommended urine checks after deciding against prophylactic steroids, risking delayed detection of initially asymptomatic nephritis. This describes a potential consequence of incomplete counseling, not an observed patient outcome.

The question-specific checklist was formalized during manuscript revision, after the original clinical evaluation. Its application and the completed revision scoring identified 16 omission-positive responses, replacing the earlier omission results. A positive score required an applicable absent component essential to a caregiver safety decision; partial coverage or an incorrect statement alone was insufficient. The 27 changed response codes show that the explicit revision criteria affected classification. A zero code means that no qualifying omission was identified under these rules, not that the response was completely correct or clinically safe. Similar concerns about completeness have been reported in LLM health-advice reviews and patient-education studies (13, 17, 21).

### 4.3 Relative platform performance depends on the evaluation domain

ChatGPT scored higher than Doubao on GQS and mDISCERN, whereas DeepSeek had the highest median QAMAI score and exceeded Doubao on that measure; no single platform was superior across all quality instruments. Prior cross-platform studies in pediatric anesthesia, peritoneal dialysis, hypertension, and oral lichen planus have similarly shown that relative performance may vary by assessment instrument (17–20), and comparable domain-dependent differences have been observed in dental and urologic patient-education studies (23, 31). In a separate warfarin-counseling study, ChatGPT and Gemini did not differ significantly in accuracy, while ChatGPT scored higher in scientific adequacy and Gemini in clarity (33). This provides a cross-setting example of distinct dimensions producing different comparisons, although its models, clinical topic, and scoring tools differ from ours. GQS emphasizes overall quality and usefulness, mDISCERN focuses on reliability and source transparency, and QAMAI evaluates AI-generated medical information across accuracy, clarity, relevance, completeness, source provision, and usefulness (22). Reliance on a single global quality instrument may therefore provide an incomplete characterization of patient-facing LLM performance.

### 4.4 Readability remains a major limitation for patient education

Responses from all three platforms were difficult to read overall, consistent with recent patient-education studies in pediatric anesthesia, prostate cancer, orthopedics, and colorectal cancer (17, 21, 29, 32). Previous studies have shown that explicit low-reading-level prompts or regeneration can improve some readability metrics (24, 26, 27), although comparisons with expert-authored materials and dermatology research suggest that the magnitude and consistency of improvement are variable (25, 28). Although none of the six readability metrics showed a significant overall platform effect in this study, Bonferroni-adjusted pairwise comparisons indicated lower reading-grade or text-complexity scores for DeepSeek than for ChatGPT on ARI, FKGL, CLI, and SMOG. These findings do not support a broad claim that one platform is consistently superior across all readability measures, but they suggest metric-specific differences. For patient education, improvements in medical information quality should therefore be accompanied by deliberate control of sentence length, terminology density, and reading level.

### 4.5 Understandability and actionability should be evaluated separately

DeepSeek had the highest PEMAT-U score, whereas ChatGPT had the highest PEMAT-A score, indicating that being able to understand information is not equivalent to being able to act on it. Recent studies in hypertension, dentistry, and urology have similarly shown that understandability and actionability may diverge (19, 23, 31), and research on patient instructions has emphasized that explicit action steps represent a dimension distinct from linguistic readability (30). For IgAV, actionable patient education should not only explain the disease but also specify when medical attention is needed, what parameters require monitoring, and how follow-up should be conducted (3–5, 8); gastrointestinal warning symptoms should likewise be paired with clear instructions for urgent evaluation (10).

### 4.6 Clinical implications

LLM platforms may serve as adjunctive tools for pediatric IgAV patient education, but they should not currently replace professional medical guidance. Recent patient-education and health-advice studies emphasize the need to consider content reliability, transparency, and output stability in patient-facing LLM applications (11, 13, 15), while readability and actionability are also important determinants of practical use (19, 31). Given the clinical features of pediatric IgAV, future applications should strengthen guideline concordance, emphasize warning signs and renal follow-up (3–5), provide explicit care-seeking advice for gastrointestinal risk (10), and retain professional medical oversight.

Differences among platforms may arise from multiple factors, including the underlying model, system prompts, retrieval or search functions, model routing, and product design. Therefore, this study reflects the overall information-service performance experienced by ordinary users in real-world product environments rather than isolated performance differences between foundation models. For patient education, such end-to-end evaluation is more closely aligned with real-world use.

### 4.7 Strengths and limitations

The study compared the same 32 standardized English questions across platforms, used a multidimensional evaluation framework, and quantified agreement between the original clinical reviewers using ICC(2,1). The revision adds an explicit question-specific checklist, response-level evidence, and element-level coding to make the omission analysis traceable. The research team completed the revision scoring and confirmed the final omission results. The checklist was established during revision and was not prespecified before the original evaluation.

Several limitations should be considered. First, only ChatGPT, DeepSeek, and Doubao were evaluated, and only the first generated response to each question was retained; response stability across repeated generations was not assessed. Second, reviewers were not blinded to platform identity, which may have introduced observer bias. Third, the final question set was predominantly guideline derived, while Google Trends and social media were used mainly for needs validation and limited supplementation; thus, the distribution may not fully represent spontaneous questions asked by patients and caregivers (34, 35), introducing possible question-selection bias. Fourth, differences in response length across platforms may affect completeness, quality, readability, and PEMAT scores, and potential confounding by response length was not fully excluded. Fifth, automated readability metrics and expert-rated instruments cannot fully substitute for the understanding, trust, or behavioral responses of actual caregivers. Sixth, the three platform contrasts were adjusted within each outcome, but no familywise correction was made across the 11 outcomes; isolated findings should therefore be interpreted as exploratory. Seventh, all platforms were evaluated in their default operating environments at the time of data collection; between-platform differences may therefore reflect a combination of foundation models, retrieval augmentation, system prompts, and product design and cannot be attributed solely to the underlying model. Eighth, generative-AI platforms evolve rapidly, and these findings represent only the August 9, 2026, data-collection time point rather than a stable long-term ranking. Finally, only English questions and English responses were evaluated, and generalization to other languages and cultural contexts should be cautious.

The revised omission analysis has additional limitations. Its checklist was formalized after the original evaluation and is not a validated instrument. Element selection and safety thresholds involve study-specific judgments. Initial coding used a tool from the same provider as one evaluated platform, creating the possibility of provider-related evaluation bias. The research team confirmed the final scoring, but a new independent two-reviewer procedure, blinded reassessment, and interrater reliability estimate were not documented for the revised omission codes. The other deficiency categories retain their original ratings and were not comprehensively rescored. The findings are descriptive and must not be interpreted as demonstrating that ChatGPT responses were omission-free in general or safer than the other platforms.

## 5 Conclusions

At the evaluated data-collection time point and under default user conditions, ChatGPT, DeepSeek, and Doubao showed domain-specific differences in pediatric IgAV patient education. No hallucinations were identified in this sample, but clinically relevant information omissions remained an important safety concern. Platforms differed in quality, reliability, understandability, and actionability, and their responses generally imposed a substantial reading burden. These findings represent platform-level, end-to-end output performance and should not be interpreted as a direct ranking of underlying foundation-model capabilities. LLM chatbot platforms may provide supplementary patient-education information, but professional medical review and clinical oversight remain necessary before use for individualized health guidance.

## Data Availability

The original contributions presented in the study are included in the article/supplementary material, further inquiries can be directed to the corresponding author.
